# The efficacy and safety of tacrolimus monotherapy in adult-onset nephrotic syndrome caused by idiopathic membranous nephropathy

**DOI:** 10.1080/0886022X.2017.1325371

**Published:** 2017-05-31

**Authors:** Qian Liang, Heng Li, Xishao Xie, Fangzhi Qu, Xiayu Li, Jianghua Chen

**Affiliations:** Kidney Disease Center, The First Affiliated Hospital, College of Medicine, Zhejiang University, Hangzhou, China

**Keywords:** Tacrolimus, idiopathic membranous nephropathy, nephrotic syndrome, immunosuppression, Chinese patients

## Abstract

**Introduction:** The purpose of the study is to evaluate the efficiency and safety of tacrolimus (TAC) monotherapy in the treatment of nephrotic idiopathic membranous nephropathy (IMN) compared with the protocol of cyclophosphamide (CTX) combined with corticosteroids.

**Methods:** In total, 58 patients with nephrotic syndrome and biopsy-proven IMN were included in this study. 30 patients received TAC monotherapy with an initial dose of 0.05–0.1 mg/kg/day. 28 patients received transvenous CTX at a dose of 0.5–0.75 g/m^2^ once in every month initially for 6 months and once in every 2 or 3 months for the later period, and the regimen was combined with corticosteroids (prednisone 1 mg/kg/d). All patients were observed for the treatment effects, recurrence and side effects.

**Results:** Twelve months after the initial treatment, a total of 24 (80%) patients in the TAC group and 23 (82.1%) patients in the CTX group achieved remission (either partial or complete remission). The survival curve of the probability of remission and complete remission were similar between the two groups (*p* > .05). Proteinuria (based on 24 h urinary protein excretion) was significantly decreased, and serum albumin was significantly increased after immunosuppressive treatment in both the groups. Estimated glomerular filtration rate (eGFR) was comparable between before and after treatment. The main adverse effects in TAC treatment were glucose intolerance, diabetes and abnormal aminotransferase.

**Conclusions:** TAC monotherapy is an alternative therapeutic regimen for patients with nephrotic IMN. Its short-term efficiency and patient tolerance are both acceptable.

## Introduction

Idiopathic membranous nephropathy (IMN) is one of the major causes of adult-onset nephrotic syndrome [1]. Among IMN patients, approximately 30–40% develops end-stage renal disease (ESRD) within 10–15 years [2]. The Kidney Disease: Improving Global Outcomes (KDIGO) guideline recommends that initial therapy with immunosuppressive agents can be started only in patients with nephrotic syndrome whose urinary protein excretion persistently exceeds 4 g/day, remains at over 50% of the baseline value, and does not exhibit a progressive decline during antihypertensive and antiproteinuric therapy during an observation period of at least 6 months (1B) [3].

Immunosuppressive treatment of IMN remains a matter of debate. The KDIGO contends that for IMN patients with nephrotic syndrome, a 6-month course with alternating monthly cycles of corticosteroids and alkylating agents (cyclophosphamide or chlorambucil) is the treatment approach best supported by evidence (1B) [3,4]. Previous studies have reported that the regimen of cyclophosphamide (CTX) and steroids induces remissions in a high proportion of patients, arrests progression of renal insufficiency and improves quality of life [5–7]. However, serious adverse effects of this standard therapy, including serious infection, myelosuppression and gonadoinhibitory effects, limited their clinical applications [8,9].

Calcineurin inhibitors (CNI), including cyclosporine (CsA) and tacrolimus (TAC), were recommended as alternative regimens for initial therapy of nephrotic IMN (1 C) [3]. In contrast with CsA, TAC exhibited a stronger immunosuppressive effect and fewer side effects in transplantation therapy [10,11]. A number of randomized controlled trials (RCTs) have reported that TAC combined with corticosteroids exhibited a satisfactory effect compared with CTX plus corticosteroids [12–14]. However, corticosteroids can still cause adverse effects, such as infection, femoral head necrosis, central obesity and liver function damage. In 2007, Praga et al. found that TAC monotherapy was effective in the treatment of IMN, increasing the probability of remission in 82% of patients after 12 months of treatment compared with 24% in the control group [15]. However, the main weakness with this trial was that it only included conservative therapies as the control group. Furthermore, the period from onset of illness to treatment was long. These features have been questioned.

The purpose of the study is to evaluate the efficiency and safety of TAC monotherapy in the treatment of nephrotic IMN compared with the protocol of CTX combined with corticosteroids.

## Methods

### Patients

This prospective cohort study was performed at a single centre, the Kidney Disease Center of the First Affiliated Hospital, College of Medicine, Zhejiang University, from January 2013 to April 2016. The ethics committee of our hospital (Medical Ethics Committee of the First Affiliated Hospital, College of Medicine, Zhejiang University) approved the study protocol and all patients were informed written consent.

The inclusion criteria were as follows: (1) age between 18 and 75 years; (2) confirmed as the onset IMN by renal biopsy in our centre; (3) with nephrotic syndrome, which was defined as urinary protein excretion of ≥3.5 g/24 h, and serum albumin of ≤30 g/L; (4) initial serum creatinine level of <133 μmol/L; and (5) no immunosuppressive agents used in the previous 6 months. The exclusion criteria were as follows: (1) secondary membranous nephropathy, such as systemic lupus erythematosus; (2) malignant tumour; (3) infection, such as hepatitis B or C virus infection, tuberculosis and syphilis; (4) fasting blood glucose >6.2 mmol/L; (5) pregnancy or lactating and (6) coexistence of life-threatening complications, such as heart failure or active gastrointestinal bleeding.

### Treatment

Patients assigned to the CTX group received transvenous CTX and oral prednisone. CTX was intravenously administered at a dose of 0.5–0.75 g/m^2^ once in every month for the initial 6 months and once in every 2–3 months for the later period. The accumulated dosage was 150 mg/kg. Oral prednisone was administered at a dose of 1 mg/kg/d for 4 weeks and tapered 5 mg every 2 weeks to 30 mg/d and then reduced 5 mg every 4 weeks until complete withdrawal at the end of 12 months. Patients assigned to the TAC group received TAC at an initial dose of 0.05–0.1 mg/kg/day divided into two doses at intervals of 12 h without corticosteroids. The dose was adjusted according to the target trough blood concentration of 5–10 ng/ml for the first 6 months and reduced from 4 to 6 ng/ml for the subsequent 3 months. Then, the dose was tapered gradually and discontinued at the end of 12 months. TAC dosage should be reduced by 30% when ≥30% increase in serum creatinine is noted compared with the baseline value, and TAC is withdrawn if the renal function was not improved after 2 weeks. The total treatment duration was 12 month for both the groups.

Calcium-channel blockers (CCBs), β-receptor blockers and diuretics were prescribed in those patients who did not meet the target blood pressure (< 125/75 mmHg). To avoid the influence of angiotensin-converting enzyme inhibitors (ACEIs) and angiotensin II subtype 1 receptor blockers (ARBs) on urinary protein, both drugs were not initiated during immunosuppressive therapy but were continued in patients who already received ACEI or ARB before recruitment. Altiazem was used to elevate the concentration of TAC in blood. Anticoagulant drugs and statins were prescribed to all the patients.

Patients were requested to come back to hospital on a weekly basis for the first month and once in every month thereafter. The following variables were measured at baseline and during observation time: blood pressure, a complete blood count, urinalysis, serum creatinine (SCr, μmol/L), estimated glomerular filtration rate [eGFR ml/min/1.73 m^2^ = 175 × Scr^−1.234^ × Age^−0.197^ (if female, × 0.79)] [16], 24-h urinary protein excretion (g), serum albumin (g/L), glucose (mg/dl), lipid profile and uric acid (μmol/L). After the initiation of TAC treatment, TAC trough blood levels were assessed weekly and stable levels of TAC were achieved. Then, the levels were assessed monthly.

### End points and definitions

The primary end point of the trial was the remission rate, including complete remission (CR) and partial remission (PR). CR was defined as a daily proteinuria level <0.5 g with stable renal function. PR was defined as proteinuria of 0.5–3.5 g/d that was reduced no less than 50% of baseline levels with well-preserved renal function. Total remission (TR) was defined as either CR or PR. Moreover, no remission (NR) was defined as patients who did not achieve CR or PR criteria after 6 months of initial treatment. Relapse was defined as proteinuria >3.5 g/d in two consecutive urinalyses or a persistent severe hypoproteinaemia in patients who had achieved CR or PR.

The secondary end points included changes in proteinuria, serum albumin, eGFR and side effects.

### Statistical analysis

Continuous variables are reported as the mean ± standard deviation (SD), and categorical variables are represented as numbers and percentages. Differences of quantitative parameters between groups were compared using the independent *t* test (for normally distributed data) or nonparametric test (for non-normally distributed data). Differences of qualitative data were estimated by chi-square test. Survival curves were analyzed using Kaplan–Meier curves, and differences were assessed by the log-rank test. *p* Values < .05 was considered as significant, and all *p* values were two-sided. Statistical analyses were performed using SPSS (version 22).

## Results

In total, 58 patients with biopsy-proved IMN of recent onset were assigned to the TAC group (*n* = 30) or CTX group (*n* = 28). All patients assigned to the TAC group completed the treatment protocol, and CTX was temporarily withheld in two patients due to leucopenia and serious pneumonia. The median follow-up period after cessation of immunosuppressive treatment was 10 months (0.2–18 months) in the TAC group and 10.5 months (0.3–19 months) in the CTX group. The demographic, laboratory, histological features and concomitant medications at baseline are presented in Table 1. No difference was observed between the two groups. All patients had nephrotic-range proteinuria with normal renal function.

**Table 1.** Baseline data of patients in the TAC and CTX group.

	TAC (*n* = 30)	CTX (*n* = 28)	*p*
Age (years)	48.2 ± 13.5	53.9 ± 10.4	.08
Gender (male/female)	16/14	9/19	.08
BMI	24.1 ± 3.0	24.9 ± 4.5	.46
Systolic pressure (mm Hg)	124.3 ± 16.0	129.9 ± 16.3	.21
Diastolic pressure (mm Hg)	76.4 ± 11.9	81.9 ± 13.2	.12
Serum creatinine (μmol/L)	70.7 ± 17.5	81.0 ± 22.5	.07
eGFR (ml/min)	93.6 ± 21.7	87.9 ± 24.9	.07
24h urinary protein (g)	5.9 ± 2.7	6.9 ± 2.2	.99
Serum albumin (g/l)	26.5 ± 6.2	24.1 ± 6.2	.15
Triglyceride (mmol/l)	2.7 ± 1.8	3.1 ± 2.3	.45
Total cholesterol (mmol/l)	7.5 ± 2.0	8.8 ± 3.0	.07
Uric acid (μmol/L)	342.8 ± 107.9	349.4 ± 79.6	.79
ALT (U/L)	14.4 ± 1.8	14.9 ± 1.6	.95
Blood glucose	5.2 ± 0.3	5.0 ± 0.5	.75
Histology grading of IMN			.39
Stage I	20	19	
Stage II	9	9	
Stage III	1	1	
Stage IV	0	0	
Concomitant medications			
Calcium-channel blockers	3	5	.39
β-receptor blockers	1	2	.51
Diuretics	2	1	.59
ACEI and/or ARB	9	7	.67

BMI: Body Mass Index; eGFR: estimated glomerular filtration rate; ALT: alanine aminotransferase; IMN: idiopathic membranous nephropahthy; ACEI: angiotensin-converting enzyme inhibitors; ARB: angiotensin II subtype 1 receptor blockers.

### Primary end points

**Figure 1.**
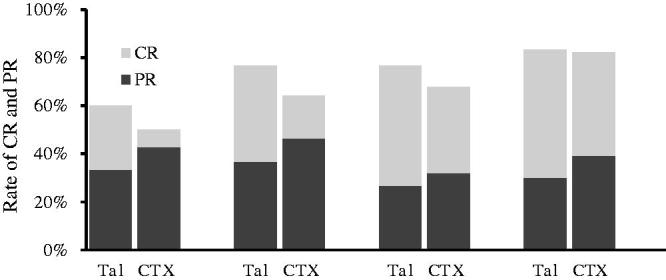
Percentages of remission (either partial or complete remission) in the TAC and CTX groups during the 12 months of therapy. The remission rates were similar between the two groups (*p* > .05).

**Figure 2.**
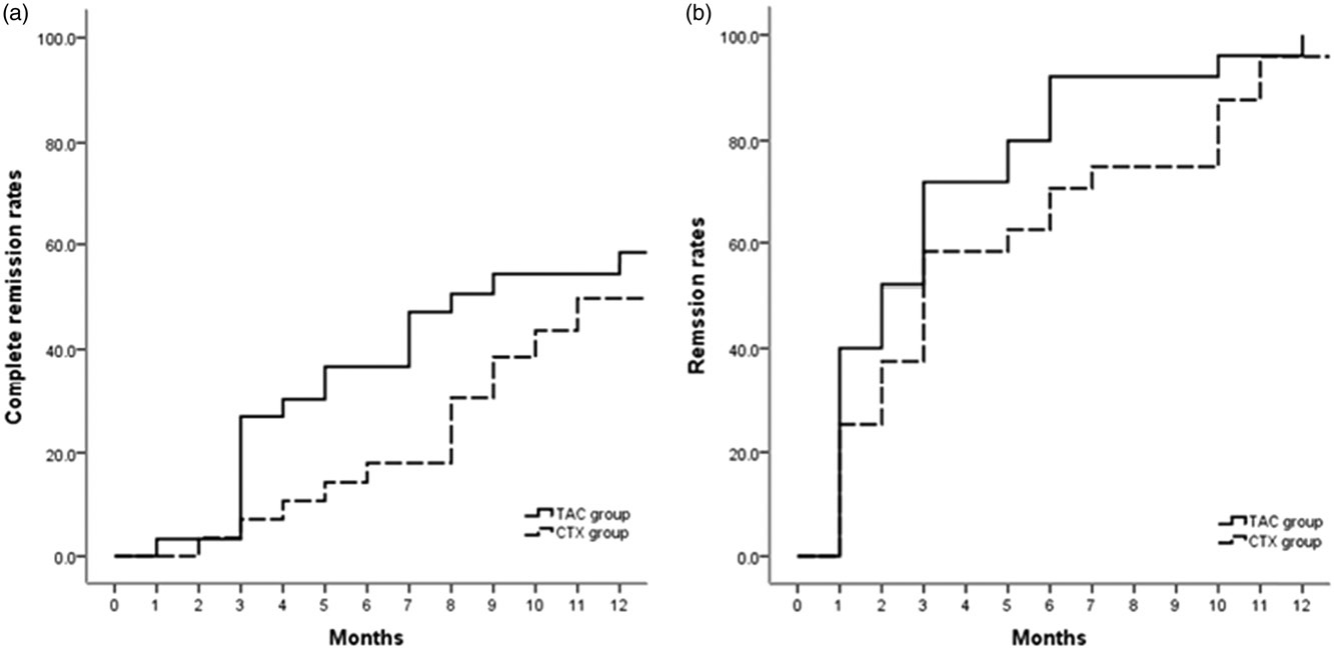
Probability of remission (either partial or complete remission) (log-rank test *p* = .62) and complete remission alone (log-rank test *p* = .36) in the TAC and CTX groups.

The CR and PR in both groups are presented in Figure 1. After 6 months of initial therapy, 12 (40%) and 5 (17.9%) cases achieved CR in the TAC and CTX groups, respectively (*p* = .06). At 12 months, 15 (50%) and 12 (42.9%) patients experienced CR in the TAC and CTX groups, respectively (*p* = .7). PR was observed in 11 (36.7%) patients in the TAC group and 13 (46.4%) patients in the CTX group at 6 months (*p* = .5) as well as 9 (30%) patients in the TAC group and 11 (39.3%) patients in the CTX group at 12 months (*p* = .5). Thus, a total of 24 (80%) patients in the TAC group and 23 (82.1%) patients in the CTX group achieved TR at 12 months after initial treatment (*p* = .6). The survival curve of the probability of remission (either CR or PR) and CR were similar between two groups (Figure 2). The average time to remission excluding the NR patients was 3.2 ± 2.9 months (range 1–18 months) in the TAC group and 5.0 ± 4.4 months (range 1–24 months) in the CTX group (*p* = .1).

No recurrence was reported in the CTX group. Three patients (10%) in the TAC group suffered relapse after attaining CR. Two of them relapsed after complete TAC withdrawal by month 12, and the remaining patient relapsed at 24 months. Relapsing patients received the original plan of TAC monotherapy. Two of these patients obtained remission of nephrotic syndrome again, and another patient switched to another immunosuppressive regimen due to poor effect in a few months. The relapse rate in the TAC group was increased compared with the CTX group.

### Secondary end points

**Figure 3.**
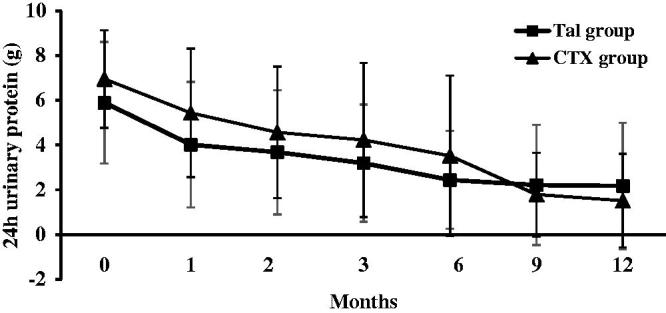
Changes of 24-h urinary protein excretion (mean ± SD) during the 12 months of therapy in the TAC and CTX group.

At 12 months after initial therapy, the 24 h urinary protein decreased from 5.9 ± 2.7 g to 2.2 ± 2.9 g in the TAC group (*p* < .01) and from 6.9 ± 2.2 g to 1.5 ± 2.1 g in the CTX group (*p* < .01). The reduction in proteinuria was similar between two groups (65.8 ± 10.9% in TAC group and 76.7 ± 8.7% in CTX group, *p* > .05). There was no statistically significant difference in the level of urinary protein excretion between the two groups at all observation times (*p* > .05) (Figure 3).

**Figure 4.**
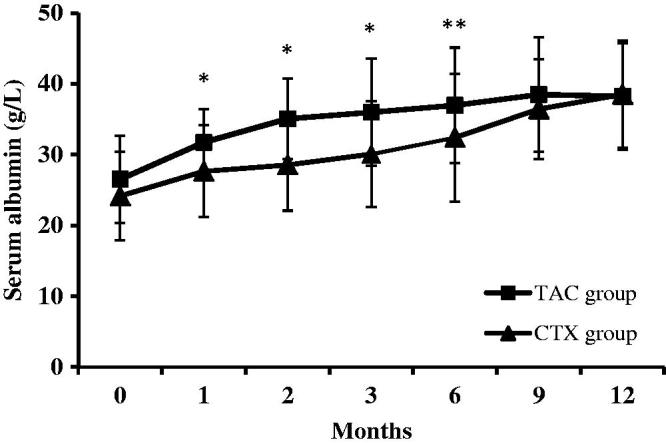
Changes of serum albumin levels (mean ± SD) during the 12 months of therapy in the TAC and CTX group. *p values < .05 compared to the CTX group; **p values < .01 compared to the CTX group.

The level of serum albumin increased from 26.5 ± 6.2 g/L to 36.9 ± 8.2 g/L in the TAC group (*p* < .01) and from 24.1 ± 6.2 g/L to 32.4 ± 9.0 g/L in the CTX group (*p* < .01) at the end of 12 months. The serum albumin level in the TAC group was significantly increased compared with that of the CTX group at 1 to 6 months (*p* < .01 at 1–3 months, *p* < .05 at 4–6 months), whereas there was no significant difference between the two groups from 7 to 12 months (*p* > .05) (Figure 4).

**Figure 5.**
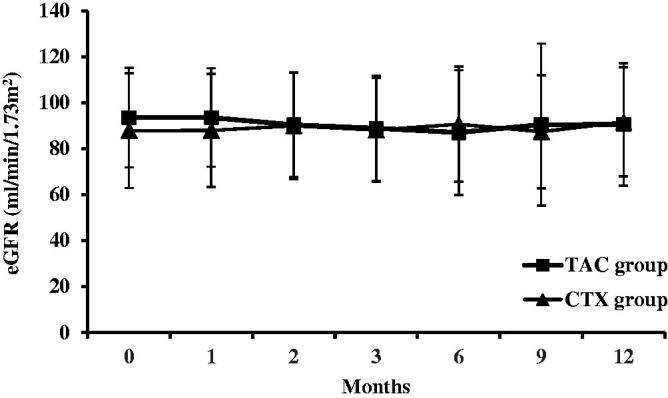
Changes of eGFR (mean ± SD) during the 12 months of therapy in the TAC and CTX group.

The eGFR was comparable before and after treatment in both the groups (93.6 ± 21.7 mL/min/1.73 m^2^ versus 90.6 ± 26.7 mL/min/1.73 m^2^ in the TAC group, *p* > .05; 87.9 ± 24.9 mL/min/1.73 m^2^ versus 91.7 ± 23.8 mL/min/1.73 m^2^, *p* > .05). In addition, no difference was noted between the two groups at any time point (*p* > .05) (Figure 5). One patient received a decreased dosage of TAC due to increasing serum creatinine.

### Blood concentration of TAC

In patients who achieved CR or PR, the effective mean trough blood level and daily dosage of TAC were 5.3 ± 1.8 ng/ml and 3.9 ± 2.8 mg/d during the first 6 months therapy. The blood concentration of TAC was 5.8 ± 1.6 ng/ml in CR patients, that in PR patients was 4.8 ± 2.1 ng/ml, and that in NR patients was 3.1 ± 1.1 ng/ml. There was significant difference between CR and NR patients (*p* < .05) (Table 2).

**Table 2.** Blood concentration of TAC (mean ± SD).

	Cases	Blood concentration (ng/ml)
CR patients	12	5.8 ± 1.6*
PR patients	11	4.8 ± 2.1
NR patients	7	3.1 ± 1.1

Compared with NR patients.

* *p* < .05.

### Side effects

Adverse effects are displayed in Table 3. No patient died or progressed to ESRD during the follow-up. A total of 11 patients (4 in the TAC group and 7 in the CTX group) developed glucose intolerance, 5 of them improved after diet control and exercise, other patients relieved after drug (TAC or corticorsteroids) reduction. Six patients (three in the TAC group and three in the CTX group) had diabetes. All of them received oral antidiabetic drugs or insulin treatment, and blood glucose level in two patients returned to normal after TAC withdrawal. Eight patients (two in the TAC group and six in the CTX group) had hepatic dysfunction, and recovered after treated with liver protective drugs. Seven patients experienced gastrointestinal symptoms such as nausea and vomiting, but the side effects were mild and endurable. Nine patients had urinary tract infection, and the incidence in TAC group (3.3%) was significantly lower than that in CTX group (28.6%) (*p* < .01). One patient in CTX group suffered from severe pulmonary infection, and recovered after anti-infection therapy in hospital. TAC was well-tolerated.

**Table 3.** The side effects in the TAC and CTX groups.

	TAC group	CTX group	*p*
Leucopenia	1 (3.3%)	2 (7.1%)	.51
Anaemia	1 (3.3%)	6 (21.4%)	.06
Abnormal aminotransferase	2 (6.7%)	6 (21.4%)	.10
Hypertension	0	0	
Cardiovascular events	0	0	
Diabetes	3 (10%)	3 (10.7%)	.93
Glucose intolerance	4 (13.3%)	7 (17.9%)	.53
Menstrual disorder	0	0	
Gastrointestinal symptoms	2 (6.7%)	5 (17.9%)	.07
Acute renal failure	0	0	
Urinary tract infection	1 (3.3%)	8 (28.6%)	.01*
Pneumonia	0	1	.30
Malignant tumour	0	0	

* *p* values < .05 compared to the CTX group.

## Discussion

A sustained remission of nephrotic syndrome is an acceptable alternative to the overall effect in the treatment of IMN [3]. Sustained remission may be achieved by means of reducing proteinuria, protecting renal function and minimizing complications. Immunosuppressive therapy can increase the remission rate of patients with IMN [17]. Early treatment can reduce the complication associated with nephrotic syndrome and can prevent progression to ESRD [18]. In addition, long-term use of immunosuppressive agents increased the potential for adverse outcomes [19]. Thus, immunosuppressive therapy in patients with IMN should be balanced against the benefit and risks [3]. In our study, a similar remission rate was observed for the CTX regimens and TAC monotherapy patients, which was followed by less frequent adverse reactions.

Recently, IMN has been considered a type of glomerular damage to the membrane antigen component of the podocyte that is mediated by autoantibodies [20]. TAC stabilizes the podocyte cytoskeleton by inhibiting the expression of transient receptor potential cation channel 6 (TRPC6) protein and the expression of calcineurin, thereby reducing the level of urinary proteins [21,22]. TAC induces remission of nephrotic syndrome in a high proportion (80%) of IMN patients. This finding was similar to previous studies [12,15]. Furthermore, there was no significant difference in the remission rate compared with the TAC plus corticosteroids regimen [12–14]. This finding is clinically relevant, considering IMN affects predominantly middle aged and elderly patients who are particularly prone to the adverse effects and infectious complications of glucocorticoids [23,24].

We found similar remission rate in the two study groups. Proteinuria was significantly decreased, and serum albumin was gradually increased after immunosuppressive treatment in both the groups. Of note, in the treatment from 1 to 6 months, TAC ameliorated hypoalbuminaemia more effectively than CTX, but no significant difference was observed after 6 months, suggesting that TAC might be more efficient in the early stage of the treatment. This finding may be related to the promotion of liver regeneration by TAC, which attributed to an inhibition of IL-2 production as well as natural killer cell activity [25]. At the same time, we found that the clinical efficacy of TAC in the treatment of IMN is promising when the plasma concentration is within the range of 5.3 ± 1.8 ng/ml.

A large number of studies demonstrated that IMN has a tendency to relapse after CNI withdrawal or tapering, and the relapse rate ranged from 13% to 50% [12,13]. In the study of Praga et al. as mentioned above, the incidence of relapse after withdrawal was 47%, with no difference in the placebo group [15]. Our study observed that the probability of relapse after remission with TAC monotherapy was 10%, which was lower than that reported in previous studies. This result may be limited by a short follow-up duration and withdrawal bias. Recent evidence suggested that prolonged TAC treatment with a low blood concentration (0.5 mg twice daily for 12–24 months) can alleviate the nephrotic syndrome persistently and maintain a low recurrence rate [26].

CNI-related nephrotoxicity is an important issue of concern, which has limited its clinical applications. Numerous studies have attempted to investigate the renal toxicity induced by CNIs. Surveys, such as those conducted by Cattran (2007) and du Buf (2004), demonstrated that CsA therapy reduces the probability of renal insufficiency [27,28]. An RCT in 2010 performed repeat renal biopsies to verify whether the TAC has renal toxicity, and no typical signs of nephrotoxicity of CNI were detected [14]. In the present study, no significant increase in serum creatinine or decreased eGFR was observed during TAC therapy. However, patients enrolled in this study had stable renal function before treatment. Therefore, whether TAC in patients with basic renal insufficiency can lead to renal toxicity still needs to be confirmed by further clinical trials.

Our findings suggest a favourable safety profile of TAC. The efficacy of the alkylating agent in IMN is confirmed by several RCTs [4–7], but this drug can be associated with severe side effects of infection and infertility. In this situation, it is quite difficult to evaluate the risks and benefits [29]. The main side effects of TAC-only therapy that we observed included hyperglycaemia, gastrointestinal reaction and abnormal aminotransferase. No patient changed his or her treatment regimen due to the adverse events of TAC. The effect of TAC on blood glucose has received increasing attention from clinicians. We found three patients (10%) with new-onset diabetes mellitus and four patients (13.33%) with impaired glucose tolerance taking oral TAC during follow-up, which was not different when compared with the CTX group. Remarkably, the incidence of infectious complications in the TAC group was significantly reduced compared with the CTX group. This finding is largely due to the elimination of the interference of corticosteroids.

This study has several limitations. First, most of the patients were randomly assigned to groups in our study, whereas a few patients had a strong desire to choose the regimen. Patient preference was mainly influenced by finances or insurance. Considering medical ethics, we must respect the wishes of patients. Given the relatively small sample, we did not remove these patients. Second, compared with the natural history of IMN for several decades, the follow-up time in our trial was relatively short, and the median observation period was only 12 months (6–30 months). Third, assuming a response rate of 80% with standard therapy, approximately 200 patients would need to be recruited to detect any differences with 80% statistical power and 5% difference in the study treatment. As a single-centre study, our sample size was limited. Therefore, a large, randomized selected study is needed. Fourth, studies showed that serum anti-PLA2R antibodies levels correlated with IMN activity and prognosis [30,31]. Regular detection of the titre is helpful to predict the remission of the disease and adjust the concentration of drug [32]. It is regrettable that our hospital carried out testing for anti-PLA2R antibodies in a recent year. Therefore, most patients lacked this laboratory testing results.

## Conclusions

TAC monotherapy is an alternative therapeutic regimen for patients with nephritic IMN. Its short-term efficiency is not inferior to that of the CTX combined with corticosteroid regimen, and the regimen is associated with fewer side effects. The long-term efficacy and safety of TAC needs further research.
